# Associations of maternal vitamin D, PTH and calcium with hypertensive disorders of pregnancy and associated adverse perinatal outcomes: Findings from the Born in Bradford cohort study

**DOI:** 10.1038/s41598-018-37600-9

**Published:** 2019-02-04

**Authors:** Gillian Santorelli, Donald Whitelaw, Diane Farrar, Jane West, Debbie A. Lawlor

**Affiliations:** 1 Bradford Institute for Health Research, Temple Bank House, Bradford Royal Infirmary, Bradford, BD9 6RJ UK; 2 Department of Diabetes and Endocrinology, Bradford Royal Infirmary, Bradford, BD9 6RJ UK; 3 MRC Integrated Epidemiology Unit at the University of Bristol, Rm OS11, Oakfield House, Oakfield Grove, Bristol, BS8 2BN UK; 4 Bristol NIHR Biomedical Research Centre, Bristol, UK

## Abstract

Vitamin D and parathyroid hormone (PTH) regulate mineral metabolism and are required to maintain calcium levels. Vitamin D deficiency is common, particularly during pregnancy, and has been associated with hypertensive disorders of pregnancy. We sought to determine whether maternal 25(OH)D, PTH and calcium concentrations at 26 weeks gestation are associated with adverse outcomes of pregnancy and establish whether these differ by ethnicity. This study included 476 White British and 534 Pakistani origin mother-offspring pairs from the Born in Bradford cohort study. We used multinomial or logistic regression to explore the association between vitamin D, PTH and calcium with gestational hypertension (GH), pre-eclampsia (PE), caesarean section (CS), preterm birth (PTB) and small for gestational age (SGA). Pakistani women had lower 25(OH)D (median 13.0 vs 36.0 nmol/L), higher PTH (median 7.7 vs 3.3 pmol/L) and similar calcium concentrations compared to White British women. In Pakistani women, higher concentrations of 25(OH)D were associated with a 60% increased odds of GH, and a 37% reduced odds of SGA; PTH was associated with a 45% reduction in the odds of GH. In White British women, each 1 SD increase in calcium concentration was associated with a 34% increase in developing GH but a 33% reduction in the odds of PTB. Associations with PE and CS were consistent with the null. In conclusion, there are ethnic differences in the associations of 25(OH)D, PTH and calcium with important perinatal outcomes. Future research would benefit from examining the associations of 25(OH)D, PTH and calcium together with a range of perinatal outcomes in order to assess the risk-benefit action of each.

## Introduction

Low vitamin D levels are a global public health problem^1^. Vitamin D can be obtained from foods (in the form of 25(OH)D_2_) or dietary supplements, but the main source for most people is synthesised in the skin from sunlight exposure containing sufficient ultraviolet B (UVB) radiation (25(OH)D_3_). At latitudes above 37°N, there is insufficient sunlight-induced vitamin D synthesis from October to late March; it is therefore essential to obtain vitamin D from dietary or supplementary sources during the winter months of northern countries, including the UK. People with darker skin are at particular risk of low vitamin D levels, and a number of studies have shown that South Asian’s are known to have lower levels of 25-hydroxyvitamin D [25(OH)D] compared to the White population^2–4^.

Vitamin D deficiency is commonly observed in pregnancy^1^ and has previously been associated with an increased risk of hypertensive disorders of pregnancy (gestational hypertension and pre-eclampsia)^1,5–9^, preterm birth^10–14^, small for gestational age (SGA) infants^14–21^ and caesarean birth^22–24^. Together with parathyroid hormone (PTH), vitamin D is required for the regulation of calcium^25^; however, despite their close interactions, the association between them and adverse perinatal outcomes is still not fully understood. Although calcium supplementation during pregnancy seems to reduce the risk of high blood pressure, pre-eclampsia and preterm birth^26^, the effects of vitamin D supplementation are less clear. A recent systematic review found evidence of an increase in mean birth weight and a reduced risk of SGA in women receiving vitamin D supplementation; however, no associations with other maternal and neonatal outcomes were observed and consequently the evidence was deemed insufficient to guide practice^16^. To our knowledge no studies have examined the combined associations between vitamin D, PTH and calcium levels with hypertensive disorders of pregnancy (HDP) and associated adverse perinatal outcomes or explored whether any of these associations differ between ethnic groups. Given the known ethnic differences in distributions of pregnancy circulating 25(OH)D^27,28^, which is a biomarker for vitamin D status, it is important to establish whether ethnicity influcences associations of 25(OH)D, PTH and calcium with perinatal outcomes. This information could help determine whether thresholds for defining categories of vitamin D deficiency should differ by ethnicity.

The aims of this study are therefore to (a) determine whether maternal circulating 25(OH)D, PTH and calcium concentrations are associated with gestational hypertension and pre-eclampsia and associated adverse perinatal outcomes (caesarean section, preterm birth and small for gestational age) in White British and Pakistani women, and (b) establish whether the magnitude or direction of associations between 25(OH)D, PTH and calcium with these outcomes differ between White British and Pakistani women.

## Methods

**Figure 1:**
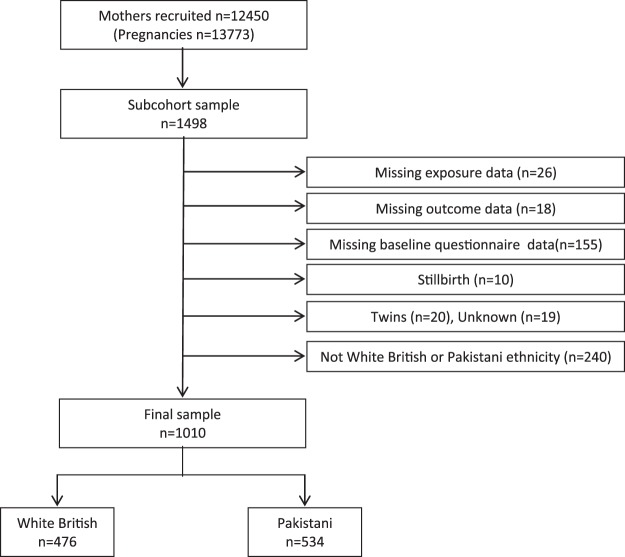
Study flow chart.

Born in Bradford (BiB) is a longitudinal multi-ethnic cohort study that was established to examine how genetic, nutrition, environmental, behavioural and social factors impact on health and development during childhood, and subsequently adult life^29^. Bradford, located at 53.8°N, is the 6^th^ largest city in the UK, is ethnically diverse, and has high levels of socio-economic deprivation. Women were recruited to BiB at approximately 26 weeks gestation when they attended for oral glucose tolerance testing (OGTT), performed as part of a universal screening programme for gestational diabetes. At recruitment, participants gave informed consent, height and weight were measured and an interview-administered baseline questionnaire completed. A total of 12,450 mothers were recruited across 13,773 pregnancies between March 2007 and December 2010, resulting in 13,858 births. Ethics approval was granted by Bradford National Health Service Research Ethics Committee (ref 07/H1302/112) on 10 March 2008, and all research was performed in accordance with relevant guidelines/regulations. A subsample of 1,498 women who attended for assessment between September 2008 and March 2009 consented to have additional blood samples analysed to assess 25(OH)D, PTH and calcium concentrations. We have previously reported findings from this cohort regarding associations between these variables and gestational diabetes, which suggested that whilst an association with 25(OH)D and PTH was not indicated, the association with calcium warranted further investigation^30^. Women were excluded from the present analyses if 25(OH)D, PTH and calcium levels were not measured (n = 26), perinatal outcomes were not reported (n = 18) and the baseline questionnaire was not completed (n = 155). Women who had a stillbirth (n = 10), a multiple birth (n = 20) or where the type of birth (singleton or multiple) was unknown (n = 19), or who were from an ethnic background other than White British or Pakistani (n = 240) were also excluded. The final sample size was 1010 (476 White British and 534 Pakistani; see Fig. 1).

### Outcome measures

We assessed associations of maternal circulating 25(OH) D, PTH and calcium (measured at 26–28 weeks gestation) with: (1) HDP: gestational hypertension (defined as new onset elevated blood pressure after 20 weeks gestation where SPB ≥140 and/or DBP ≥90 on two or more occasions), and pre-eclampsia (defined as gestational hypertension with concurrent significant proteinurea ≥1+); (2) caesarean section; (3) preterm birth (defined as gestational age at birth <37 weeks); and (4) small for gestational age (SGA), based on the 2011 UK-WHO charts^31^.

Mode of birth (vaginal or caesarean section), birthweight and gestational age were extracted from the hospital’s electronic maternity record system. Blood pressure measurements and levels of proteinurea during pregnancy were extracted from the maternity notes.

### Exposures

Our exposures were maternal pregnancy concentrations of serum 25(OH)D, plasma PTH and serum calcium. Fasting venous samples were taken at the time of the OGTT (median 26.3 weeks of gestation (IQR 26.0, 26.9).

Serum 25(OH)D samples were immediately frozen at −80 °C and analysed within 12 months with no interim thaw cycles. Serum samples were deproteinized using zinc sulphate and acetonitrile. We used deuterated 25(OH)D_3_ [hexadeuterium-25(OH)D_3_] as an internal standard in all analyses. Chromatographic separation was achieved on a Waters Sun Fire C18 column, and detection of 25(OH)D_2_ and 25(OH)D_3_ was achieved using Waters Micromass Quattro Ultima Platinum Mass Spectrometer. Between-batch coefficients of variation (CoV) were 5–8% and 4–6% for 25(OH)D_2_ and 25(OH)D_3_ respectively. As concentrations of 25(OH)D_2_ were generally very low, total 25(OH)D was calculated by summing 25(OH)D_2_ and 25(OH)D_3_ and is presented as nmol/L.

Plasma PTH and serum calcium were analysed promptly at Bradford Royal Infirmary (BRI). Chemiluminescence immunoassay was used to measure intact PTH with a manufacturer’s quoted coefficients of variation of 7.8% at 4.3 pmol/L and a detection range of 0.265 to 201 pmol/L. Calcium concentrations were measured using a colorimetric assay and were adjusted for albumin; values are presented as mmol/L.

### Confounders

We adjusted our analyses for a number of confounders with 25(OH)D levels that have been identified by previous research. Information on maternal age, ethnicity, mother’s highest educational qualifications (defined as less than A-level and A-level and above. A-levels are subject-specific qualifications taken by students aged 16–18 in England, Wales and Northern Ireland, and are the main school leaving qualifications in these countries) and in receipt of means-tested benefits (which aim to give extra support to individuals who can demonstrate that their income and capital are below a certain level) as proxy measures for socio-economic position, smoking during pregnancy, vitamin D supplementation (at least once a week) and physical activity (estimated using the General Practice Physical Activity Questionnaire (GPPAQ)^32^) were obtained from the baseline questionnaire. Early pregnancy BMI was derived from maternal height and weight measured at pregnancy booking (~12 weeks gestation). Sunshine exposure was estimated in the three months before the samples were taken using data from the Bradford station of the UK meteorological office^33,34^.

### Statistical analysis

The distribution of outcomes and risk factors for both ethnic groups are presented as frequency (percentage), mean (SD) or median (IQR). Associations of 25(OH)D, PTH and calcium with gestational hypertension, pre-eclampsia and associated adverse outcomes were assessed using multinomial or logistic regression with the exposure as a continuous variable. Two models were considered for each outcome: model 1 was unadjusted, and model 2 was adjusted for the confounders described above. The physical activity questionnaire has four categories: inactive, moderately inactive, moderately active and active; however, due to low numbers in the ‘active’ category, this was merged with ‘moderately active’ for the analysis. Associations between the exposures and outcomes are presented as odds ratios per 1 standard deviation (SD) increase. The analysis was done for all women, and for White British and Pakistani women separately and tested for any differences in associations between the ethnic groups (i.e. tested for an interaction between ethnicity and exposure). All analyses were performed using STATA/SE software (Stata/SE 13.1 for Windows, StataCorp LP, College Station, TX, USA).

### Ethics approval

Ethics approval was granted by Bradford National Health Service Research Ethics Committee (ref 07/H1302/112) on 10 March 2008.

## Results

### Overall and ethnic differences in maternal exposures with gestational hypertension, pre-eclampsia and associated adverse perinatal outcomes

**Figure 2:**
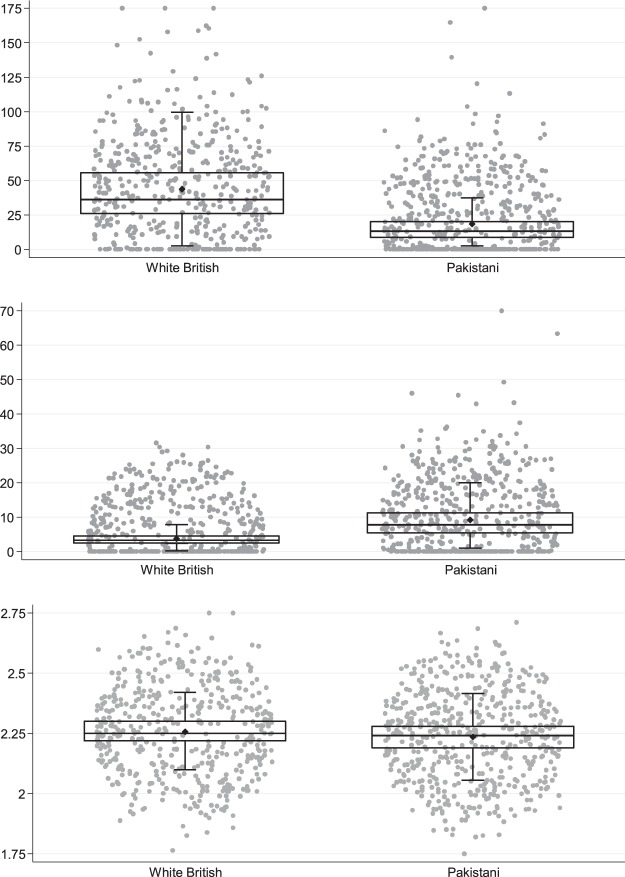
Box and scatter plots of maternal circulating 25(OH)D, PTH and calcium concentrations in White British and Pakistani women.

Table 1 shows the distribution of outcomes, exposures and maternal characteristics. Compared to White British women, Pakistani women were slightly older (27.6 vs 26.4 years), had a lower BMI (25.2 vs 27.0 kg/m^2^), were less likely to smoke during pregnancy (3.8% vs 34.2%), were more likely to be in receipt of means-tested benefits (47.2 vs 37.0%) and reported lower physical activity levels (93.3% vs 76.0% inactive or moderately inactive). On average, Pakistani women had lower median levels of 25(OH)D (13.0 vs 36.0 nmol/mL^2^) and higher levels of PTH (7.9 vs 3.3 pmol/L^2^) than White British women; both groups had similar distributions of calcium (see Fig. 2). A greater proportion of White British women developed gestational hypertension (12.0% vs 5.4%) and pre-eclampsia (3.4% vs 1.7%), and had a caesarean (22.5% vs 19.9%) or preterm birth (6.5% vs 4.5%) compared to Pakistani women. Conversely, delivery of a small for gestational age baby was more common in Pakistani women (19.9% vs 8.6%).

**Table 1:** Maternal characteristics and perinatal outcomes for all pregnancies and by ethnic group (White British and Pakistani). Values are n (%) unless otherwise indicated.

Maternal characteristics and perinatal outcomes	All N = 1010	White British N = 476	Pakistani N = 534
Age (years)^*^	27.0 ± 5.5	26.4 ± 6.3	27.6 ± 5.3
BMI at 12 weeks gestation^*^	26.0 ± 5.8	27.0 ± 6.2	25.2 ± 5.3
Smoked during pregnancy			
No	827 (81.9)	313 (65.8)	514 (96.3)
Yes	183 (18.1)	163 (34.2)	20 (3.8)
In receipt of means-tested benefits			
No	582 (57.6)	300 (62.4)	282 (52.8)
Yes	428 (42.4)	176 (37.0)	252 (47.2)
Maternal education			
Less than A level	627 (62.1)	304 (63.9)	323 (60.5)
A level and above	383 (37.9)	172 (36.1)	211 (39.5)
Physical activity level			
Inactive	668 (66.1)	242 (50.8)	426 (79.8)
Moderately inactive	181 (19.0)	120 (25.2)	72 (13.5)
Moderately active	94 (9.3)	70 (14.7)	24 (4.5)
Active	56 (5.5)	44 (9.2)	12 (2.3)
Vitamin D supplementation			
No	835 (82.7)	389 (81.7)	446 (83.5)
Yes	175 (17.3)	87 (18.3)	88 (16.5)
Sunshine, hours per 3 mths^†^	248.9 (161.9–315.7)	258.5 (164.2, 322.6)	237.7 (159.5, 308.9)
25(OH)D, nmol/mL^†^	22.6 (12.0–40.8)	36.0 (26.0–55.5)	13.0 (8.8–20.3)
Vitamin D status			
Deficient	598 (61.5)	160 (34.8)	438 (85.4)
Insufficient	196 (20.1)	152 (33.0)	44 (8.6)
Adequate	179 (18.4)	148 (32.2)	31 (6.0)
PTH, pmol/L^†^	5.0 (3.2–8.2)	3.3 (2.4–4.5)	7.9 (5.5–11.3)
Calcium, mmol/L^†^	2.25 (2.20–2.29)	2.25 (2.22–2.30)	2.24 (2.19–2.28)
Gestational hypertension	86 (8.5)	57 (12.0)	29 (5.4)
Pre-eclampsia	25 (2.5)	16 (3.4)	9 (1.7)
Caesarean birth	213 (21.1)	107 (22.5)	106 (19.9)
Preterm birth (<37 wks)	55 (5.5)	31 (6.5)	24 (4.5)
Small for gestational age	147 (14.6)	41 (8.6)	106 (19.9)

*Mean (SD); ^†^Median (IQR).

### Associations between 25(OH)D, PTH and calcium with gestational hypertension, pre-eclampsia and associated adverse perinatal outcomes

**Figure 3:**
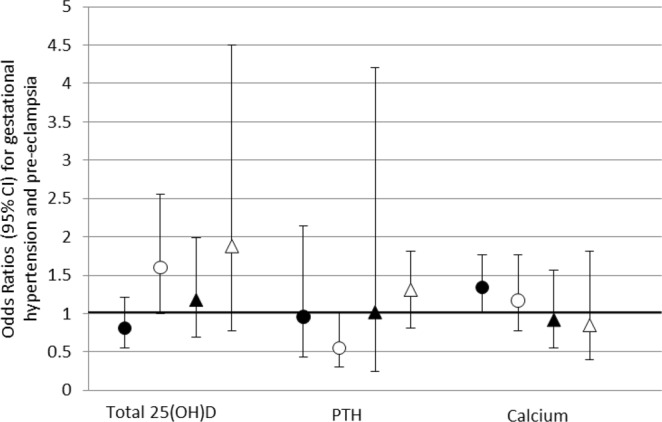
Adjusted odds ratios with 95% CI for associations between a 1 SD increase in maternal circulating 25(OH)D, PTH and calcium with hypertensive disorders of pregnancy. Gestational hypertension: ● White British women ▲ Pakistani women. Pre-eclampsia: ○ White British women △ Pakistani women.

**Figure 4:**
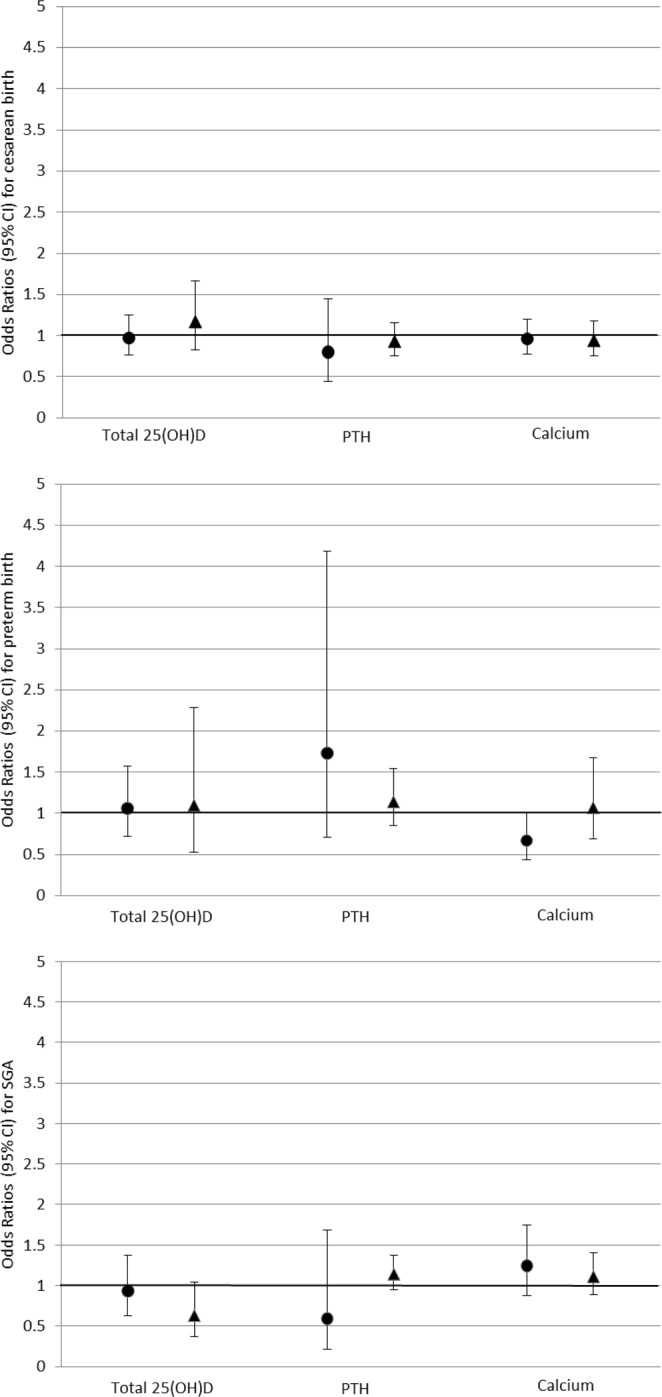
Adjusted odds ratios with 95% CI for associations between a 1 SD increase in maternal circulating 25(OH)D, PTH and calcium with caesarean section, preterm birth and small for gestational age. ● White British women ▲ Pakistani women.

Adjusted (Model 2) associations of each exposure with outcomes in White British and Pakistani mother-offspring pairs are shown in Table 2, and Figs 3 and 4, with the unadjusted (Model 1) results in the supplementary material. In the adjusted analyses, higher concentrations of 25(OH)D in Pakistani women were associated with a 60% increased odds of developing gestational hypertension, whereas higher PTH levels were associated with a 45% relative reduction, and there was evidence of a statistical interaction between the ethnic groups (p < 0.01 and p = 0.10 respectively). 25(OH)D was also associated with a reduced odds of SGA and higher calcium concentrations were associated with gestational hypertension but a reduced odds of preterm birth in White British but not Pakistani women; however, there was no strong statistical support for any ethnic differences, with p-values for interaction with ethnicity being ≥1.0. Associations with caesarean delivery were broadly consistent with the null.

**Table 2:** Adjusted associations of maternal circulating 25(OH)D, PTH and calcium with hypertensive disorders of pregnancy (HDP) and adverse associated outcomes overall and stratified by ethnicity.

Outcome	aOR^†^ (95% CI) for each outcome per 1 SD of exposure			P_Interaction_^‡^
	All (n = 978)	White British (n = 462)	Pakistani (n = 516)	
**HDP** ^*^				
25(OH)D				
Gestational hypertension	1.2014 (0.94–1.54)	0.81 (0.55–1.21)	1.60 (1.00–2.55)	<0.001
Pre-eclampsia	1.33 (0.91–1.94)	1.18 (0.69–1.99)	1.88 (0.78–4.50)	0.58
PTH				
Gestational hypertension	0.55 (0.37–0.83)	0.96 (0.43–2.14)	0.55 (0.30–1.01)	0.10
Pre-eclampsia	1.05 (0.66–1.66)	1.02 (0.25–4.21)	1.31 (0.81–2.11)	0.87
Calcium				
Gestational hypertension	1.35 (1.08–1.21)	1.34 (1.01–1.76)	1.17 (0.78–1.76)	0.70
Pre-eclampsia	0.92 (0.60–1.39)	0.92 (0.55–1.56)	0.85 (0.40–1.81)	0.95
**Caesarean birth**				
25(OH)D	1.02 (0.86–1.21)	0.97 (0.76–1.25)	1.17 (0.83–1.66)	0.31
PTH	0.93 (0.77–1.11)	0.80 (0.44–1.45)	0.93 (0.75–1.16)	0.99
Calcium	0.94 (0.80–1.10)	0.906(0.77–1.20)	0.94 (0.75–1.18)	0.77
**Preterm birth**				
25(OH)D	1.16 (0.86–1.55)	1.06 (0.72–1.57)	1.10 (0.53–2.28)	0.78
PTH	1.11 (0.86–1.45)	1.73 (0.71–4.18)	1.07 (0.85–1.54)	0.60
Calcium	0.84 (0.63–1.12)	0.67 (0.44–1.01)	1.07 (0.69–1.68)	0.12
**Small for gestational age**				
25(OH)D	0.58 (0.44–0.77)	0.93 (0.63–1.37)	0.63 (0.37–1.04)	0.37
PTH	1.30 (1.10–1.53)	0.59 (0.21–1.68)	1.14 (0.95–1.37)	0.31
Calcium	1.08 (0.90–1.30)	1.24 (0.88–1.74)	1.11 (0.89–1.40)	0.67

*Hypertensive disorders of pregnancy; normotensive is the reference category.

^†^Adjusted for maternal age, BMI, highest educational attainment, smoking status in pregnancy, in receipt of benefits, physical activity levels, hours of sunshine and vitamin D supplementation.

^‡^P-value for interaction with ethnicity.

## Discussion

### Main findings

We found that at 26 weeks gestation, White British women had markedly higher levels of 25(OH)D levels, lower PTH and similar calcium levels compared to Pakistani women. We found evidence for ethnic differences in associations between maternal 25(OH)D and PTH with gestational hypertension. Where point estimates differed between the two groups, the directions of these with respect to clinical outcomes were inconsistent. For example, our results suggest higher average 25(OH)D could be detrimental to Pakistani women by increasing their risk of gestational hypertension but beneficial in reducing their risk of SGA. Higher calcium concentrations were associated with an increased risk of gestational hypertension but reduced the risk of preterm birth in White British women.

### Strengths and limitations

The strengths of our study include its prospective bi-ethnic cohort design, direct measurement of 25(OH)D, PTH and calcium levels and detailed information on a number of potential confounders. Limitations are that due to overall small numbers, and hence relatively few cases for the outcomes examined, we have limited statistical power, especially when considering ethnic differences, however our study is able to show potential areas where investigation should be directed. Related to the relatively small sample sizes we were unable to explore possible non-linear associations of 25(OH)D, PTH and calcium with our outcomes. It is known that concentrations of 25(OH)D fluctuate over time; however we were only able to assess it once, at around 26 weeks gestation. It would have been desirable to have also had data at earlier and later time-points in order to evaluate change over time, as it is possible that concentrations at different stages of fetal development or that change during pregnancy is important in the development of adverse perinatal outcomes. Due to a lack of diet-related data, we were not able to adjust for this in our analyses. However, we have directly measured total plasma 25(OH)D concentration, and have accounted for sunshine exposure and vitamin D supplementation in the analyses. As few foods are naturally rich in vitamin D, it is unlikely that including diet in our analyses would have had an impact. Our results are from White British and Pakistani origin women resident in the UK and may therefore not be generalisable to other White or South Asian ethnic groups. However, in stratifying our analyses by ethnic group we were able to observe the effect of the exposures on outcomes in the majority ethnic group in the UK (White British) and in Pakistani-origin women, who account for a significant proportion of the population in a number of UK towns and cities.

### Interpretation

There is growing evidence that low levels of 25(OH)D can adversely affect cardiometabolic health in adults including associations with both hypertension and insulin resistance^35,36^. In pregnancy, there is evidence to suggest an association between 25(OH)D and the pathophysiology of pre-eclampsia^8^. One recent study reported similar 25-hydroxyvitamin D3 levels in normotensive pregnant women and women with pre-eclampsia, but higher levels of vitamin D metabolites and impaired placental uptake of 25-hydroxyvitamin D3 in women with pre-eclampsia^37^, suggesting vitamin D metabolism may influence physiological processes in a number of ways.

Pre-eclampsia is associated with increased maternal and infant mortality and morbidity therefore interventions to ameliorate these risks urgently required. Vitamin D supplementation to improve outcomes including reducing the risk of pre-eclampsia continues to attract investigation. A recent systematic review included 43 trials and identified a further 35 registered trials, unfortunately only eight of the 43 included trials were considered at low risk of bias^16^. Conflicting with data from previous observational studies showing an inverse relationship with vitamin D levels and pre-eclampsia, we demonstrated a positive association and the review of Vitamin D supplementation found no evidence of an effect on risk of pre-eclampsia or gestational hypertension. Reasons for inconsistencies between trials and observational studies investigating associations are unclear, but may include: the possibility that there are different effects associated with naturally occurring vitamin D synthesised from sunlight compared to vitamin D taken as a supplement; different effects from natural or artificial vitamin D metabolites; heterogeneity of included trials in the review and variation in the definition of pre-eclampsia across trials or effects from variables not measured in observational studies. Our study and the findings of two other reviews share similarities in that we found higher concentrations of 25(OH)D reduced the risk of SGA^16,17^ and an increase in mean birth weight^16^ with supplementation. Low vitamin D may lead to low calcium levels which may in turn lead to increased vasoconstriction and hypertension by stimulating PTH or renin release, and calcium has previously been shown to be inversely related to pre-eclampsia^38^. Strong evidence from high quality trials suggests a beneficial effect on high blood pressure and pre-eclampsia from calcium supplementation (at least 1 g/day) in pregnancy, but an increase in the risk of HELLP (haemolysis, elevated liver enzymes and low platelets) syndrome; the authors suggest this latter inconsistent finding may result from the control of blood pressure without treatment of the underlying pre-eclamptic process^26^. Conversely we found a positive association between calcium and gestational hypertension, which may be a result of small numbers and consequently few events but may also be due to the relatively small change in each SD of calcium in unsupplemented women in our study compared to the large differences in calcium levels from supplementation.

## Conclusion

Our findings indicate ethnic differences exist in the associations between 25(OH)D, PTH and calcium with HDP and associated adverse perinatal outcomes, and that the directions of these associations were inconsistent with respect to clinical importance. The potential combination for benefit and harm for different outcomes highlights the importance of examining several related exposure and outcomes in one study.

## Supplementary information

Supplementary table 1

## Data Availability

Data can be made available upon request.
