# Supplementary material for: Associations of maternal vitamin D, PTH and calcium with hypertensive disorders of pregnancy and associated adverse perinatal outcomes: Findings from the Born in Bradford cohort study
Source: Sci Rep. 2019 Feb 4;9:1205. doi: 10.1038/s41598-018-37600-9 (PMC6362043; doi:10.1038/s41598-018-37600-9)
Supplement: Supplementary file 1 — Supplementary table 1 [file 41598_2018_37600_MOESM1_ESM.pdf]

Associations of maternal vitamin D, PTH and calcium with hypertensive disorders of pregnancy and associated adverse perinatal outcomes: Findings from the Born in Bradford cohort study

Gillian Santorelli, Donald Whitelaw, Diane Farrar, Jane West, Debbie A. Lawlor

S1: Unadjusted associations of maternal circulating 25(OH)D, PTH and calcium with hypertensive disorders of pregnancy (HDP) and associated adverse outcomes overall and stratified by ethnicity.

	OR (95% CI) for each outcome per 1 SD of exposure			$P_{Interaction}$ †
**Outcome**	**All (n=1010)**	**White British (n=476)**	**Pakistani (n=534)**	
**HDP***				
25(OH)D				
Gestational hypertension	1.16 (0.95 - 1.42)	0.74 (0.53 - 1.01)	1.58 (1.08 - 2.30)	<0.01
Pre-eclampsia	1.36 (0.99 - 1.86)	1.11 (0.71 - 1.73)	1.53 (0.80 - 2.81)	0.42
PTH				
Gestational hypertension	0.60 (0.42 - 0.83)	1.43 (0.75 - 2.74)	0.57 (0.34 - 0.96)	0.03
Pre-eclampsia	0.90 (0.58 - 1.39)	1.11 (0.31 - 3.97)	1.16 (0.75 - 1.80)	0.96
Calcium				
Gestational hypertension	1.58 (1.27 - 1.95)	1.57 (1.21 - 2.03)	1.35 (0.91 - 2.00)	0.54
Pre-eclampsia	1.07 (0.71 - 1.60)	1.02 (0.61 - 1.71)	1.00 (0.50 - 1.97)	0.54
**Caesarean birth**				
25(OH)D	1.09 (0.94 - 1.26)	1.00 (0.81 - 1.24)	1.21 (0.91 - 1.61)	0.29
PTH	0.91 (0.77 - 1.07)	1.06 (0.61 - 1.84)	0.91 (0.75 - 1.12)	0.62
Calcium	0.99 (0.85 - 1.15)	0.94 (0.76 - 1.17)	1.02 (0.82 - 1.27)	0.63
**Preterm birth**				
25(OH)D	1.08 (0.83 - 1.39)	1.01 (0.71 - 1.44)	0.85 (0.42 - 1.72)	0.67
PTH	1.05 (0.82 - 1.35)	1.25 (0.53 - 2.99)	1.20 (0.91 - 1.57)	0.92
Calcium	0.85 (0.65 - 1.11)	0.68 (0.46 - 0.99)	1.04 (0.68 - 1.59)	0.13
**Small for gestational age**				
25(OH)D	0.58 (0.45, 0.75)	0.82 (0.58 - 1.17)	0.63 (0.41 - 0.97)	0.35
PTH	1.29 (1.12, 1.50)	0.47 (0.17 - 1.31)	1.13 (0.95 - 1.34)	0.10
Calcium	0.99 (0.83 - 1.18)	1.11 (0.81 - 1.51)	1.05 (0.84 - 1.30)	0.76

* Hypertensive disorders of pregnancy; normotensive is the reference category

† P-value for interaction with ethnicity
